# Second primary malignancies in patients with male breast cancer

**DOI:** 10.1038/sj.bjc.6602505

**Published:** 2005-03-29

**Authors:** K Hemminki, G Scélo, P Boffetta, L Mellemkjaer, E Tracey, A Andersen, D H Brewster, E Pukkala, M McBride, E V Kliewer, K-S Chia, V Pompe-Kirn, C Martos, J G Jonasson, X Li, P Brennan

**Affiliations:** 1 Division of Molecular Genetic Epidemiology, German Cancer Research Center (DKFZ), Im Neuenheimer Feld 580, Heidelberg D-69120, Germany; 2 Department of Biosciences at Novum, Karolinska Institute, Huddinge, Sweden; 3 International Agency for Research on Cancer (IARC), Lyon, France; 4 Institute of Cancer Epidemiology, Danish Cancer Society, Copenhagen, Denmark; 5 Central Cancer Registry, Woolloomooloo, New South Wales, Australia; 6 The Cancer Registry of Norway, Oslo, Norway; 7 Scottish Cancer Registry, Information Services, NHS National Services Scotland, Edinburgh, Scotland; 8 Finnish Cancer Registry, Institute for Statistical and Epidemiology Cancer Research, Helsinki, Finland; 9 Cancer Control Research Programme, British Columbia Cancer Registry, Vancouver, BC, Canada; 10 Epidemiology and Cancer Registry, CancerCare Manitoba, Winnipeg, Canada; 11 Community Health Sciences, University of Manitoba, Winnipeg, Canada; 12 Singapore Cancer Registry, Singapore; 13 Cancer Registry of Slovenia, Institute of Oncology, Ljubljana, Slovenia; 14 Cancer Registry of Zaragoza, Health Department of Aragon Government, Zaragoza, Spain; 15 Icelandic Cancer Registry, Icelandic Cancer Society, Reykjavik, Iceland; 16 Faculty of Medicine, University of Iceland, Reykjavik, Iceland

**Keywords:** subsequent malignancy, cancer registry, pooled analysis, BRCA in men, discordant sites

## Abstract

An international multicentre study of first and second primary neoplasms associated with male breast cancer was carried out by pooling data from 13 cancer registries. Among a total of 3409 men with primary breast cancer, 426 (12.5%) developed a second neoplasia; other than breast cancer, a 34% overall excess risk of second primary neoplasia, affecting the small intestine (standardised incidence ratio, 4.95, 95% confidence interval, 1.35–12.7), rectum (1.78, 1.20–2.54), pancreas (1.93, 1.14–3.05), skin (nonmelanoma, 1.65, 1.16–2.29), prostate (1.61, 1.34–1.93) and lymphohaematopoietic system (1.63, 1.12–2.29). A total of 225 male breast cancers was recorded after cancers other than breast cancer, but an increase was found only after lymphohaematopoietic neoplasms. *BRCA2* (and to some extent *BRCA1*) mutations may explain the findings for pancreatic and prostate cancers. Increases at other sites may be related to unknown factors or to chance. This large study shows that the risks for second discordant tumours after male breast cancer pose only a moderate excess risk.

It is well established that women with a first primary breast cancer run a 2–5-fold increased risk of developing second primary breast cancer compared with the risk for the first primary breast cancer (Chen *et al*, 1999; Vaittinen and Hemminki, 2000). In men, the risk of second breast cancer after a first breast cancer is much higher, of the order of 100 (Dong and Hemminki, 2001; Auvinen *et al*, 2002), despite the overall pathology, natural history and hormonal risk factors for sporadic and familial breast cancers showing certain similarities for both genders; socioeconomic risk factors also appear similar for male and female breast cancers (Hsing *et al*, 1998; Hemminki and Li, 2003; Hemminki *et al*, 2003b). Klinefelter syndrome, gynaecomastia and testicular disease are additional male risk factors (Hultborn *et al*, 1997; Lynch *et al*, 1999). Heritable mutations predisposing to male breast cancer include *BRCA2*, *BRCA1* and, possibly, the androgen receptor gene (Lynch *et al*, 1999; Liede *et al*, 2004). Accounting for some 15% of all cases, *BRCA2* mutations are the most common heritable factors in male breast cancer, and, in addition to breast cancer, carriers have an increased risk of prostate, pancreatic and stomach cancers and melanomas (Liede *et al*, 2004). *BRCA1* mutations are less common in male than female breast cancer, and also affect prostate cancer (Liede *et al*, 2004). Few studies have assessed the risk of second cancers, other than breast cancer, among male breast cancer patients and melanoma appears to the be the only tumour showing an excess among men (Auvinen *et al*, 2002; Hemminki and Granstrom, 2002).

In the present study, the risks of second primary neoplasms for first male breast cancer, and for male breast cancer following any other cancer are based on pooled data from 13 large cancer registries, which are collaborating in studies on second primary neoplasms. As a result, the study is two times larger than the previous largest study (Auvinen *et al*, 2002). Since concordant (breast–breast) first and second primaries are not registered in all centres, we do not report on such cancers.

## SUBJECTS AND METHODS

An international multicentre study of second primary cancers has been initiated among second primary cancers. It is a collaboration of large cancer registries operating for at least 25 years including British Columbia, Manitoba and Saskatchewan (Canada), Singapore, Slovenia, Norway, Denmark, Scotland, New South Wales (Australia), Sweden, Finland, Iceland and Zaragoza (Spain). Data were provided from each cancer registry on all initial primary cancers including age and sex of the subject, diagnosis and date of the first primary, follow-up for mortality and date and diagnosis of the second primary, if any. Information was also obtained from each cancer registry on the set of rules used for defining a second primary cancer. As these differ between cancer registries, and also over time, the International Association of Cancer Registries (IACR)/International Agency for Research on Cancer (IARC) rules on second primary cancers were adopted as a common set of rules (Muir and Percy, 1991). Tumours were included according to the recording practice of the participating centres, for example, including histologically benign tumours of the central nervous system and carcinoids at any sites. Nonmelanoma skin cancer includes 53.9% basal and 46.1% squamous cell carcinomas and other tumours; as second cancers, the proportion of these types is 49.6 and 50.4%, respectively. This was possible as all participating cancer registries currently use the IACR/IARC rules, or a local set of more extensive or detailed rules. The Swedish data have been used in a previous study of male breast cancer (Dong and Hemminki, 2001).

The follow-up time varied according to the available data from the different registries. The follow-up time was longest, 1943–1997, for the Danish and shortest, 1978–1998, for the Spanish data. Observed numbers of male neoplasms were compared to the expected number derived from the age, sex and calendar period-specific cancer incidence rates in each of the cancer registries. Standardised incidence ratios (SIR) adjusted for age, year and registry were calculated using indirect standardisation methods. Exact confidence intervals (CI) around the SIR were calculated assuming a Poisson distribution for the observed number of neoplasms. As a second type of analysis, we have calculated the SIR for male breast cancer as a second primary after all other cancer sites as a first primary.

## RESULTS

The numbers of men diagnosed with a first primary breast cancer are shown in Table 1 by various characteristics. A total of 3409 men was diagnosed, and of these 426 (12.5%) subsequently developed a second primary neoplasm. More than half of all men were over 65 years at diagnosis, as many as 25% were diagnosed before year 1975, and the most common follow-up period was 1–4 years (41.1% of all men).

The SIRs for second neoplasms by length of follow-up are shown in Table 2 for sites for which at least four cases were recorded in the overall analysis. In the overall analysis, all malignancies were increased by 34%. The highest SIRs were found for small intestine (4.95) and myeloid leukaemia (3.42); among small intestinal tumours, two were carcinoids, one was carcinoma and another one was histologically unspecified. Other specific sites of increased SIRs were the rectum (1.78), pancreas (1.93), (nonmelanoma) skin (1.65) and prostate (1.61). Nonmelanoma skin tumours were in excess only in the short follow-up period, <12 months after breast cancer; liver cancer and leukaemias were increased only in the middle follow-up period (1–9 years); pancreatic cancer was only increased in the longest follow-up period (10+ years). Only prostate cancer was increased in two follow-up periods.

The effect of diagnostic age for breast cancer on the risk of subsequent neoplasms was analysed in Table 3. Stomach, pancreatic and lung cancers were in excess among relatively young breast cancer patients (diagnosed below age 56 years); larynx cancer was increased in those diagnosed between 56 and 65 years. The SIR for small intestinal cancer was 11.5 in males diagnosed at 66–74 years. Rectal and skin cancers were increased in old breast cancer patients. For leukaemias, the histological type changed by diagnostic age of breast cancer: myeloid leukaemia was in excess in younger patients, whereas among subjects aged >75 years, lymphoid leukaemia was seen in excess.

The effect of the calendar period of breast cancer diagnosis is shown in Table 4. The overall risk of second primary neoplasms was somewhat increasing throughout the study period from 1.29 (before year 1975) to 1.41 (1991+). An excess of lung cancer was limited to patients diagnosed at 1991+, whereas skin tumours were detected in those diagnosed in the early years of follow-up.

A total of 225 male breast cancers was recorded after any other neoplasms (data not shown). No increase was found after any individual site; after all lymphohaematopoietic neoplasms, the risk for breast cancer was 1.80 (*N*=18, 1.07–2.84). An excess of breast cancer was diagnosed less than 12 months after lip cancer (SIR 5.37, *N*=3, 1.11–15.7). Breast cancer was also increased 1–9 years after prostate cancer (SIR 1.45, *N*=48, 1.07–1.92) and after multiple myeloma (SIR 3.77, *N*=4, 1.03–9.65). There was no overall increase in breast cancer after prostate cancer (SIR 1.19, *N*=56, 0.90–1.54).

## DISCUSSION

In order to interpret the results of the present study, we need to consider the treatment options that have been available throughout the study period. In general, therapies for male breast cancer have been adopted from female breast cancer, and surgery and radiotherapy, usually in combination, have been the main treatments throughout the study period (Buzdar, 2003; Gennari *et al*, 2004) with chemotherapy increasing towards the end.

The present data should be of high quality, as they were carefully controlled by the participating, well-established cancer registries. These pooled data are the largest data set used for the study of male breast cancer. Nevertheless, chance associations consequent on the multiple comparisons may be present and the findings should be considered tentative.

The finding that two cancers were increased after each other, irrespective of the order would represent suggestive evidence. For example, non-Hodgkin's lymphoma is increased after squamous cell carcinoma of the skin in men and women, and a similar association has been observed in the reverse order (Hemminki *et al*, 2003a). Such a broad internal control is, however, usually possible only for reasonably common malignancies of both genders with good survival and reasonably similar age distributions. In the present study, prostate cancer was increased after breast cancer, and breast cancer was increased after prostate cancer; however, only in one follow-up period. *BRCA2* (and to some extent *BRCA1*) mutations could explain these findings. Among other *BRCA2*-related male tumours, pancreatic cancer showed a high risk after breast cancer (overall SIR 1.93) and stomach cancer was increased in men who were diagnosed with breast cancer before the age of 56 years; however, no increase was observed for melanoma in contrast to findings from the SEER study (Auvinen *et al*, 2002; Hemminki and Granstrom, 2002).

Radiotherapy and many chemotherapy agents cause DNA damage, which may be related to the risk of subsequent neoplasms. A trend with an increasing relative risk with follow-up time would provide evidence for a relation between therapy for the primary cancer and the risk of a second cancer in the present study (Swerdlow *et al*, 2000). However, there was no increasing relative risk with follow-up time for any neoplasm following male breast cancer. Leukaemia risks were above unity throughout the follow-up periods, and they were highest for myeloid leukaemia. The only significant risk for pancreatic cancer was in the last follow-up period, but the SIRs were above unity throughout the follow-up periods; besides, therapy-induced pancreatic cancer is probably rare (Swerdlow *et al*, 2000). An increased risk of nonmelanoma skin cancer was only noted in the first follow-up period, which may be due to surveillance effects or immunological disturbances.

The sites at which increased overall SIRs were noted and which were not related to the above conditions included the small intestine and rectum. Increased SIRs were additionally noted for liver, lung and laryngeal cancers and for multiple myeloma, when a specific follow-up period or diagnostic age was considered. There are no obvious common environmental risk factors for these neoplasms. Some of the small intestinal tumours were carcinoids and these types of tumours are often diagnosed incidentally and more often among individuals with high socioeconomic status (Hemminki and Li, 2001). The involvement of sites in the same organ systems, gastrointestinal tract and respiratory system may add some credibility to the findings.

The relative increase in all second neoplasms (breast excluded) was a moderate 1.34, in marked contrast to second breast cancer, which can be up to 100 (Auvinen *et al*, 2002; Hemminki and Granstrom, 2002). However, as male breast cancers are rare, the modest excess risk of second prostate cancer may amount to equally many excess cases as second breast cancer. Excess cases are also found for pancreatic and nonmelanoma skin tumours and for leukaemias.

## Figures and Tables

**Table 1:** Characteristics of men diagnosed with first primary breast cancer

	**Second cancer status^a^**					
	**Free from second cancer**		**Have a second cancer**		**Total**	
	***n***	**%**	***n***	**%**	***n***	**%**
*Age at first cancer registration (years)*						
<56	617	20.7	60	14.1	677	19.9
56–65	706	23.7	101	23.7	807	23.7
66–74	799	26.8	152	35.7	951	27.9
75+	861	28.9	113	26.5	974	28.6

*Calendar period at first cancer registration*						
<1975	740	24.8	111	26.1	851	25
1975–1983	721	24.2	144	33.8	865	25.4
1984–1990	661	22.2	102	23.9	763	22.4
1991+	861	28.9	69	16.2	930	27.3

*Follow-up period*						
Less than 1 year	524	17.6	73	17.1	597	17.5
1–4 years	1248	41.8	153	35.9	1401	41.1
5–9 years	670	22.5	111	26.1	781	22.9
10+ years	541	18.1	89	20.9	630	18.5

Total	2983	100	426	100	3409	100

a Cancer different from the first cancer.

**Table 2:** SIR for second malignancy after first male breast cancer by length of follow-up period

	**All**			**Less than 12 months**			**1–9 years**			**10+ years**		
**Cancer sites (ICD 9th revision)**	**Observed**	**SIR**	**95% CI**	**Observed**	**SIR**	**95% CI**	**Observed**	**SIR**	**95% CI**	**Observed**	**SIR**	**95% CI**
All malignant (140–208)	426	**1.34**	(1.22–1.47)	73	**1.62**	(1.27–2.03)	264	**1.31**	(1.16–1.48)	89	1.25	(1.01–1.54)
Oral cavity, pharynx (140–149)	8	0.93	(0.40–1.82)	1	0.78	(0.02–4.34)	7	1.25	(0.50–2.57)	0		
Stomach (151)	22	1.09	(0.68–1.65)	4	1.24	(0.34–3.18)	12	0.92	(0.48–1.61)	6	1.53	(0.56–3.34)
Small intestine (152)	4	**4.95**	(1.35–12.7)	1	8.56	(0.21–47.7)	2	3.92	(0.47–14.2)	1	5.54	(0.14–30.9)
Colorectal (153, 154)	55	**1.35**	(1.02–1.76)	10	1.73	(0.83–3.19)	38	1.47	(1.04–2.02)	7	0.77	(0.31–1.58)
Colon (153)	25	1.05	(0.68–1.55)	4	1.19	(0.33–3.05)	17	1.13	(0.66–1.80)	4	0.75	(0.20–1.91)
Rectum (154)	30	**1.78**	(1.20–2.54)	6	2.48	(0.91–5.40)	21	**1.96**	(1.21–3.00)	3	0.80	(0.16–2.33)
Liver, gallbladder, bile ducts (155–156, excluding 155.2)	6	1.07	(0.39–2.33)	0			6	1.70	(0.62–3.70)	0		
Liver (155, excluding 155.2)	6	1.85	(0.68–4.02)	0			6	**2.95**	(1.08–6.41)	0		
Pancreas (157)	18	**1.93**	(1.14–3.05)	4	2.97	(0.81–7.59)	8	1.35	(0.58–2.65)	6	**2.95**	(1.08–6.43)
Larynx (161)	7	1.80	(0.73–3.72)	1	1.73	(0.04–9.66)	4	1.59	(0.43–4.08)	2	2.53	(0.31–9.16)
Lung (162)	63	1.26	(0.96–1.61)	9	1.21	(0.55–2.30)	39	1.21	(0.86–1.65)	15	1.44	(0.81–2.37)
Melanoma of skin (172)	9	1.29	(0.59–2.45)	0			7	1.58	(0.64–3.26)	2	1.24	(0.15–4.46)
Other neoplasm of skin (173)	36	**1.65**	(1.16–2.29)	9	**2.92**	(1.34–5.54)	20	1.47	(0.90–2.27)	7	1.38	(0.56–2.85)
Prostate (185)	119	**1.61**	(1.34–1.93)	19	**1.94**	(1.17–3.02)	78	**1.69**	(1.34–2.11)	22	1.23	(0.77–1.87)
Bladder (188, 189.3, 189.4)	18	0.86	(0.51–1.36)	6	2.04	(0.75–4.45)	9	0.68	(0.31–1.29)	3	0.63	(0.13–1.84)
Kidney (189, excluding 189.3, 189.4)	7	0.85	(0.34–1.75)	2	1.73	(0.21–6.25)	2	0.38	(0.05–1.38)	3	1.63	(0.34–4.78)
Lymphohaematopoietic (200–208)	33	**1.63**	(1.12–2.29)	5	1.76	(0.57–4.10)	21	**1.63**	(1.01–2.50)	7	1.54	(0.62–3.18)
Lymphomas (200–202)	11	1.33	(0.66–2.37)	1	0.87	(0.02–4.84)	6	1.14	(0.42–2.47)	4	2.14	(0.58–5.48)
Non-Hodgkin's lymphoma (200, 202)	11	1.46	(0.73–2.62)	1	0.97	(0.02–5.42)	6	1.26	(0.46–2.74)	4	2.33	(0.64–5.97)
Multiple myeloma (203)	5	1.18	(0.38–2.74)	2	3.36	(0.41–12.1)	3	1.12	(0.23–3.28)	0		
Leukaemias (204–208)	17	**2.21**	(1.29–3.54)	2	1.81	(0.22–6.55)	12	**2.45**	(1.27–4.28)	3	1.78	(0.37–5.20)
Lymphoid leukaemia (204)	7	1.86	(0.75–3.83)	0			6	2.51	(0.92–5.45)	1	1.21	(0.03–6.76)
Myeloid leukaemia (205)	8	**3.42**	(1.47–6.73)	1	3.02	(0.08–16.8)	6	**3.98**	(1.46–8.67)	1	1.98	(0.05–11.0)

SIR=standardised incidence ratios; CI=confidence interval; ICD=International Classification of Diseases. Bold numerals show that 95% CI does not include 1.00.

**Table 3:** SIR for second malignancy after first male breast cancer diagnosed at different ages

	**Age at first cancer registration (years)**											
	**<56**			**56–65**			**66–74**			**75+**		
**Cancer sites (ICD 9th revision)**	**Observed**	**SIR**	**95% CI**	**Observed**	**SIR**	**95% CI**	**Observed**	**SIR**	**95% CI**	**Observed**	**SIR**	**95% CI**
All malignant (140–208)	60	**1.62**	(1.23–2.08)	101	1.18	(0.96–1.43)	152	**1.39**	(1.18–1.63)	113	**1.32**	(1.09–1.58)
Oral cavity, pharynx (140–149)	3	1.94	(0.40–5.66)	3	1.11	(0.23–3.25)	1	0.38	(0.01–2.13)	1	0.56	(0.01–3.13)
Stomach (151)	7	**3.82**	(1.54–7.87)	1	0.20	(0.00–1.11)	12	1.64	(0.85–2.87)	2	0.34	(0.04–1.21)
Small intestine (152)	1	7.67	(0.19–42.8)	0			3	**11.50**	(2.37–33.5)	0		
Colorectal (153, 154)	5	1.10	(0.36–2.56)	12	1.11	(0.57–1.94)	16	1.15	(0.66–1.86)	22	**1.93**	(1.21–2.93)
Colon (153)	1	0.40	(0.01–2.20)	5	0.82	(0.26–1.90)	9	1.10	(0.50–2.09)	10	1.44	(0.69–2.64)
Rectum (154)	4	1.97	(0.54–5.06)	7	1.49	(0.60–3.08)	7	1.21	(0.49–2.50)	12	**2.72**	(1.41–4.76)
Liver, gallbladder, bile ducts (155–156, excluding 155.2)	0			2	1.30	(0.16–4.69)	2	1.02	(0.12–3.70)	2	1.36	(0.16–4.92)
Liver (155, excluding 155.2)	0			2	2.16	(0.26–7.82)	2	1.77	(0.21–6.38)	2	2.52	(0.30–9.11)
Pancreas (157)	5	**4.66**	(1.51–10.9)	6	2.42	(0.89–5.27)	5	1.54	(0.50–3.59)	2	0.79	(0.10–2.86)
Larynx (161)	0			5	**3.66**	(1.19–8.54)	2	1.75	(0.21–6.32)	0		
Lung (162)	13	**2.03**	(1.08–3.48)	18	1.14	(0.67–1.80)	18	1.03	(0.61–1.63)	14	1.33	(0.73–2.23)
Melanoma of skin (172)	0			3	1.38	(0.29–4.04)	3	1.52	(0.31–4.45)	3	2.37	(0.49–6.93)
Other neoplasm of skin (173)	2	0.90	(0.11–3.26)	5	0.95	(0.31–2.21)	14	**2.00**	(1.09–3.35)	15	**2.07**	(1.16–3.41)
Prostate (185)	9	1.47	(0.67–2.79)	26	1.48	(0.97–2.17)	53	**1.95**	(1.46–2.56)	31	1.35	(0.92–1.92)
Bladder (188, 189.3, 189.4)	2	0.85	(0.10–3.07)	2	0.35	(0.04–1.28)	8	1.12	(0.48–2.20)	6	1.04	(0.38–2.26)
Kidney (189, excluding 189.3, 189.4)	3	2.24	(0.46–6.55)	2	0.82	(0.10–2.95)	2	0.74	(0.09–2.66)	0		
Lymphohaematopoietic (200–208)	5	1.77	(0.58–4.14)	9	1.65	(0.75–3.13)	8	1.19	(0.51–2.35)	11	**2.10**	(1.05–3.75)
Lymphomas (200–202)	2	1.45	(0.18–5.25)	2	0.86	(0.10–3.09)	5	1.89	(0.61–4.41)	2	1.03	(0.12–3.72)
Non-Hodgkin's lymphoma (200, 202)	2	1.68	(0.20–6.06)	2	0.95	(0.12–3.44)	5	2.07	(0.67–4.83)	2	1.11	(0.13–4.01)
Multiple myeloma (203)	0			0			1	0.68	(0.02–3.79)	4	3.51	(0.96–8.99)
Leukaemias (204–208)	3	3.24	(0.67–9.47)	7	**3.52**	(1.42–7.25)	2	0.77	(0.09–2.77)	5	2.31	(0.75–5.39)
Lymphoid leukaemia (204)	1	2.28	(0.06–12.7)	2	2.00	(0.24–7.23)	0			4	**3.78**	(1.03–9.68)
Myeloid leukaemia (205)	1	3.50	(0.09–19.5)	4	**6.53**	(1.78–16.7)	2	2.50	(0.30–9.03)	1	1.55	(0.04–8.65)

SIR=standardised incidence ratios; CI=confidence interval; ICD=International Classification of Diseases. Bold numerals show that 95% CI does not include 1.00.

**Table 4:** SIR for second malignancy after first male breast cancer diagnosed at different periods

	**Period at first cancer registration**											
	**<1975**			**1975–1983**			**1984–1990**			**1991+**		
**Cancer sites (ICD 9th revision)**	**Observed**	**SIR**	**95% CI**	**Observed**	**SIR**	**95% CI**	**Observed**	**SIR**	**95% CI**	**Observed**	**SIR**	**95% CI**
All malignant (140–208)	111	**1.29**	(1.06–1.55)	144	**1.35**	(1.13–1.59)	102	**1.34**	(1.10–1.63)	69	**1.41**	(1.10–1.78)
Oral cavity, pharynx (140–149)	1	0.36	(0.01–2.03)	4	1.40	(0.38–3.59)	3	1.53	(0.32–4.48)	0		
Stomach (151)	9	1.01	(0.46–1.91)	10	1.66	(0.80–3.06)	3	0.89	(0.18–2.60)	0		
Small intestine (152)	2	8.41	(1.02–30.4)	0			1	5.31	(0.13–29.6)	1	7.99	(0.20–44.5)
Colorectal (153, 154)	13	1.15	(0.61–1.96)	21	1.52	(0.94–2.33)	14	1.47	(0.80–2.46)	7	1.16	(0.47–2.39)
Colon (153)	6	0.97	(0.36–2.11)	8	0.97	(0.42–1.92)	10	1.74	(0.84–3.20)	1	0.27	(0.01–1.52)
Rectum (154)	7	1.36	(0.55–2.80)	13	**2.34**	(1.25–4.01)	4	1.05	(0.29–2.69)	6	2.53	(0.93–5.50)
Liver (155, excluding 155.2)	0			3	2.64	(0.54–7.71)	2	2.61	(0.32–9.44)	1	1.95	(0.05–10.9)
Pancreas (157)	7	2.32	(0.93–4.77)	6	1.87	(0.69–4.08)	4	2.06	(0.56–5.26)	1	0.87	(0.02–4.83)
Larynx (161)	2	1.93	(0.23–6.97)	1	0.73	(0.02–4.04)	3	3.24	(0.67–9.46)	1	1.86	(0.05–10.4)
Lung (162)	14	1.09	(0.60–1.83)	19	0.99	(0.60–1.55)	17	1.46	(0.85–2.34)	13	**1.98**	(1.05–3.39)
Melanoma of skin (172)	1	0.87	(0.02–4.85)	4	1.85	(0.50–4.72)	1	0.44	(0.01–2.47)	3	2.15	(0.44–6.30)
Other neoplasm of skin (173)	11	**2.04**	(1.02–3.66)	12	1.54	(0.80–2.69)	8	1.58	(0.68–3.11)	5	1.42	(0.46–3.32)
Prostate (185)	25	1.41	(0.91–2.08)	38	**1.67**	(1.18–2.29)	28	1.45	(0.97–2.10)	28	**2.00**	(1.33–2.90)
Bladder (188, 189.3, 189.4)	6	1.11	(0.41–2.41)	5	0.68	(0.22–1.59)	5	1.01	(0.33–2.36)	2	0.62	(0.07–2.24)
Kidney (189, excluding 189.3, 189.4)	3	1.28	(0.26–3.75)	3	1.15	(0.24–3.35)	0			1	0.79	(0.02–4.38)
Lymphohaematopoietic (200–208)	11	**2.00**	(1.00–3.58)	10	1.49	(0.71–2.74)	7	1.43	(0.58–2.95)	5	1.58	(0.51–3.70)
Lymphomas (200–202)	5	2.55	(0.83–5.95)	3	1.10	(0.23–3.23)	2	0.93	(0.11–3.35)	1	0.68	(0.02–3.81)
Non Hodgkin lymphoma (200, 202)	5	3.03	(0.98–7.06)	3	1.22	(0.25–3.55)	2	0.99	(0.12–3.59)	1	0.72	(0.02–4.03)
Multiple myeloma (203)	1	0.84	(0.02–4.66)	1	0.70	(0.02–3.93)	1	1.00	(0.02–5.55)	2	3.13	(0.38–11.3)
Leukaemias (204–208)	5	2.14	(0.70–5.00)	6	2.33	(0.86–5.07)	4	2.32	(0.63–5.95)	2	1.89	(0.23–6.84)
Lymphoid leukaemia (204)	4	3.42	(0.93–8.75)	2	1.59	(0.19–5.75)	1	1.21	(0.03–6.73)	0		
Myeloid leukaemia (205)	1	1.60	(0.04–8.93)	4	**4.85**	(1.32–12.4)	2	3.52	(0.43–12.7)	1	3.08	(0.08–17.1)

SIR=standardised incidence ratios; CI=confidence interval; ICD=International Classification of Diseases. Bold numerals show that 95% CI does not include 1.00.
